# The British Medical Association

**Published:** 1903-08-08

**Authors:** 


### The Hospital.

A Journal of

The Medical Sciences and Hospital Administration.

Vol. XXXIV.—No. 880. AUGUST 8, 1903.

### THE BRITISH MEDICAL ASSOCIATION.

The annual meeting of the British Medical Association, which has just been held at Swansea, under the presidency of Dr. Griffiths, has presented the usual combination of a festive social gathering, an assemblage of men of science bent upon receiving and imparting instruction in their useful and noble calling, and an arena for the display of the futilities of so-called medical politics and medical politicians. A noteworthy feature of the meeting was the gracious acceptance by the Prince of Wales of the position of an honorary member of the Association, an acceptance in complete harmony with the interest which his Royal Highness has invariably shown in the work of the medical profession, and which cannot fail to conduce to the honour and welfare of the great body which he has thus consented to join. According to the *British Medical Journal*, which, as the official organ of the Association, must, we presume, be relied upon as furnishing a correct account of the proceedings, the honour received a somewhat curious recognition. "At the invitation of the president," we are informed, "the *members* sang 'God Bless the Prince of Wales,' the *audience* standing meanwhile." Who were the "members," and who the "audience," we are not informed, nor do we observe in the subsequent discussions of the ethical or other sections or committees, any proposal to insist upon a musical qualification for future membership. Something of the kind would, nevertheless, appear to be practically necessary; for it is recorded that a vote of thanks to the retiring president, Mr. Whitehead, was in like manner received with musical honours, the members rising and singing "For he's a jolly good fellow." On this occasion we hear nothing of an "audience" other than the vocalists themselves; but the general conviviality of the proceeding is not uninteresting, although it hardly seems to be in perfect harmony either with scientific earnestness or with the conduct of business in public meeting. The journal does not mention the hour of meeting, but there is no reason to believe that it was at an "advanced period of the evening."

The Report of the Ethical Committee of the Association, which was adopted after a brief discussion, was curiously incomplete. It made no mention of an unwarrantable letter written by the secretary of the Committee, with or without specific instructions, to a highly distinguished London surgeon, bringing against him an unfounded charge of having given a testimonial for the purposes of advertisement, and then, in consequence of the prompt action of the recipient, withdrawn and humbly apologised for by the Chairman of the Committee. The omission was distinctly unfortunate; because it is clear that the Committee in question is not exempt from a tendency to take unjustifiable liberties even with gentlemen whose position should render them proof against its attacks, and an example of the manner in which such attacks may be dealt with could hardly fail of being useful to others whose station is less secure.

Passing away from trivialities, and from the speakers whose attention was engrossed by them, the great features of the meeting were the two principal addresses, that on Medicine by Dr. Roberts and that on Surgery by Mr. Mayo Robson, the latter embodying personal reminiscences extending over a third of a century, and the performance of two thousand operations; the former dealing with "Infective and Infectious Diseases." It is perhaps necessary to explain that Dr. Roberts limits the word infective to diseases which are known to be produced by the direct action upon the body of living pathogenic micro-organisms; and classes as infectious all which can in any manner be communicated from person to person or from some other animal to man. The distinction is no doubt a necessary and proper one; but the suggested limitation of the meaning of the former of the two words is wholly unjustifiable as a question of language. They are, if two words can be so, absolutely synonymous. The Oxford Dictionary gives for "infectious" the definition "having the quality or power of communicating disease by infection," and for "infective" "having the quality of infecting with disease or of spreading disease by infection; infectious." And these explanations are sanctioned both by etymology and by usage. We cannot, without protest, permit the "modern teaching" to which Dr. Roberts appeals, to play tricks with our mother tongue. Of the address itself it is only necessary to add that it is eminently thoughtful and suggestive, and that it will well repay attentive perusal. On the side of surgery it is needless to say that Mr. Mayo Robson appeals to an experience not only of long duration but of very exceptional extent, and his address is little more than a catalogue of the triumphs of the knife over disease, a catalogue which, from the limitations imposed by time, was almost confined to the abdominal cavity and its contents. It would, we think, have been improved by a more systematic enforcement of the lessons of caution which we believe all large experience is calculated to convey. The operating of the last few years has unquestionably shown a tendency to extremes which cannot but be regretted, and which cannot fail, if it be continued, to exert in time a reactionary influence. Dugald Stewart somewhere says that the apparent simplicity of a perfect literary style induces those who read it to think that they also could write in a similar manner. In the same way the facility which results from perfect surgical dexterity and skill has before now, we believe, been a snare to the less skilful, and has induced them to engage in surgical undertakings which, both for their own credit and for the welfare of their patients, it would have been better to avoid. We have heard now, for many years, a great deal about the triumphs of surgery; and it would be alike interesting and instructive if some future occupant of Mr. Mayo Robson's chair would tell us something about its failures and its defects.

